# The relationship between renal cell carcinoma pathological types and perirenal fat area

**DOI:** 10.1186/s12885-025-14164-2

**Published:** 2025-05-08

**Authors:** Xin Leng, Chenchao Zhou, Jiulong Wu, Hongfang Zheng, Jianliang Wang, Qiaoxing Li, Yuhua Huang, Jianhu Liu

**Affiliations:** 1 Department of Urology, The First People’s Hospital of Kunshan, Suzhou, 215300 China; 2 Department of Urology, The First Affiliated Hospital of Soochow University, Suzhou, 215006 China; 3 Department of Radiology, The First People’s Hospital of Kunshan, Suzhou, 215300 China

**Keywords:** Perirenal fat area, Renal cell carcinoma, Clear cell renal cell carcinoma, Non-clear cell renal cell carcinoma, Pathological type

## Abstract

**Introduction:**

To explore whether there is a relationship between perirenal fat area (PFA) and the pathological types of renal cell carcinoma (RCC).

**Methods:**

Two hundred ninety-seven cases of RCC patients were included in our study, which is a retrospective analysis. Based on pathological type, we divided the 297 RCC patients into two groups: the clear cell renal cell carcinoma (ccRCC) group (236 cases) and the non-clear cell renal cell carcinoma (non-ccRCC) group (61 cases). Computed tomography (CT) images at the renal vein level were used to measure PFA. A multivariate logistic regression model was employed to examine the connection between various pathological types of RCC and PFA.

**Results:**

Significant differences were observed between ccRCC and non-ccRCC patients in PFA (*P* = 0.007), contralateral PFA (*P* = 0.011), weight (*P* = 0.002), BMI (*P* < 0.001), pathological stage 1 (*P* = 0.010), and pathological stage 2 (*P* = 0.002). To study the link between pathological subtypes and PFA, a multivariate logistic regression model was employed. Stratifying patients by tumor location in the kidney, the multivariate logistic regression analysis showed that when the tumor is located outside the polar lines of the kidney (OPLK), for every 1 cm^2^ increase in PFA, the probability of developing ccRCC increases by 5% [1.05 (1.01, 1.10) *P* = 0.0153]. Furthermore, after stratifying patients by tumor location and pathological stage, it was found that in T1 stage patients with tumors located OPLK, for every 1 cm^2^ increase in PFA, the probability of developing ccRCC increases by 6% [1.06 (1.01, 1.11) *P* = 0.0300].

**Conclusion:**

When the tumor is located OPLK in T1 stage patients, PFA is positively correlated with ccRCC. Perirenal adipose tissue may be a risk factor for ccRCC.

## Introduction

Renal cell carcinoma (RCC) ranks among the deadliest cancers affecting the urinary system [1].

The incidence of RCC typically increases with age, with 70% of new cases occurring in developed countries [2]. According to global cancer statistics from 2018, RCC ranks 16 th in incidence and 17 th in mortality among malignant tumors worldwide [3]. RCC has several pathological subtypes, with clear cell renal cell carcinoma (ccRCC) being the most common, accounting for over 70%, followed by papillary carcinoma at 10–15%, and chromophobe carcinoma at 5% [4]. CcRCC is characterized by high rates of metastasis and recurrence, with a 5-year overall survival rate of up to 96% for early-stage ccRCC patients, and less than 10% for late-stage ccRCC patients [5]. Clinically identifying the differences between ccRCC and non-ccRCC is crucial for exploring the mechanisms underlying the development and progression of renal clear cell carcinoma, as well as formulating improved treatment strategies for ccRCC.

Obesity is one of the risk factors for RCC [6]. The term"obesity"denotes the excessive accumulation of adipose tissue and/or atypical distribution of body fat. Growing evidence suggests that an increase in visceral fat significantly contributes to the development and advancement of RCC [7]. Our previous study demonstrated that the visceral fat area in ccRCC patients is significantly higher compared to patients with renal angiomyolipoma with minimal fat [8]. perirenal fat is also considered visceral fat tissue and is closer to the renal tumor tissue. Therefore, the aim of our study was to analyze the relationship between perirenal fat area (PFA) and different pathologies in RCC.

## Patients and methods

A retrospective analysis was carried out on all RCC patients who underwent surgery from January 2016 to December 2020. Cases were sourced from the First Affiliated Hospital of Soochow University and the First People’s Hospital of Kunshan. The exclusion criteria included: 1) Absence of accessible abdominal CT scan images. 2) Presence of other chronic wasting diseases, such as advanced malignancies, anorexia, tuberculosis, or hyperthyroidism. 3) Patients with missing medical record data. Two expert pathologists supplied the postoperative pathology reports. The study involved 297 patients, comprising 236 with ccRCC and 61 with non-ccRCC. The research adhered to the Helsinki Declaration and received approval from the hospital's ethics board. Every patient provided informed consent prior to surgery and successfully managed the perioperative phase.

This study included the following variables: age, gender, tumor location in the kidney, tumor side, tumor size, height, weight, body mass index (BMI), perirenal fat area (PFA), contralateral perirenal fat area (cPFA), pathology, pathological staging, hypertension, and diabetes mellitus. BMI is an indicator of obesity. According to Chinese standards, obesity is defined as BMI ≥ 28 kg/m^2^, and overweight is defined as 24 ≤ BMI < 28 kg/m^2^. Quantifying the volume of perirenal fat tissue is challenging. Eckberg SE and colleagues measured PFA using CT images at the renal vein level, as the renal vein is easier to locate in CT images and shows strong consistency [9]. We also used CT images at the renal vein level to measure PFA.

**Fig. 1:**
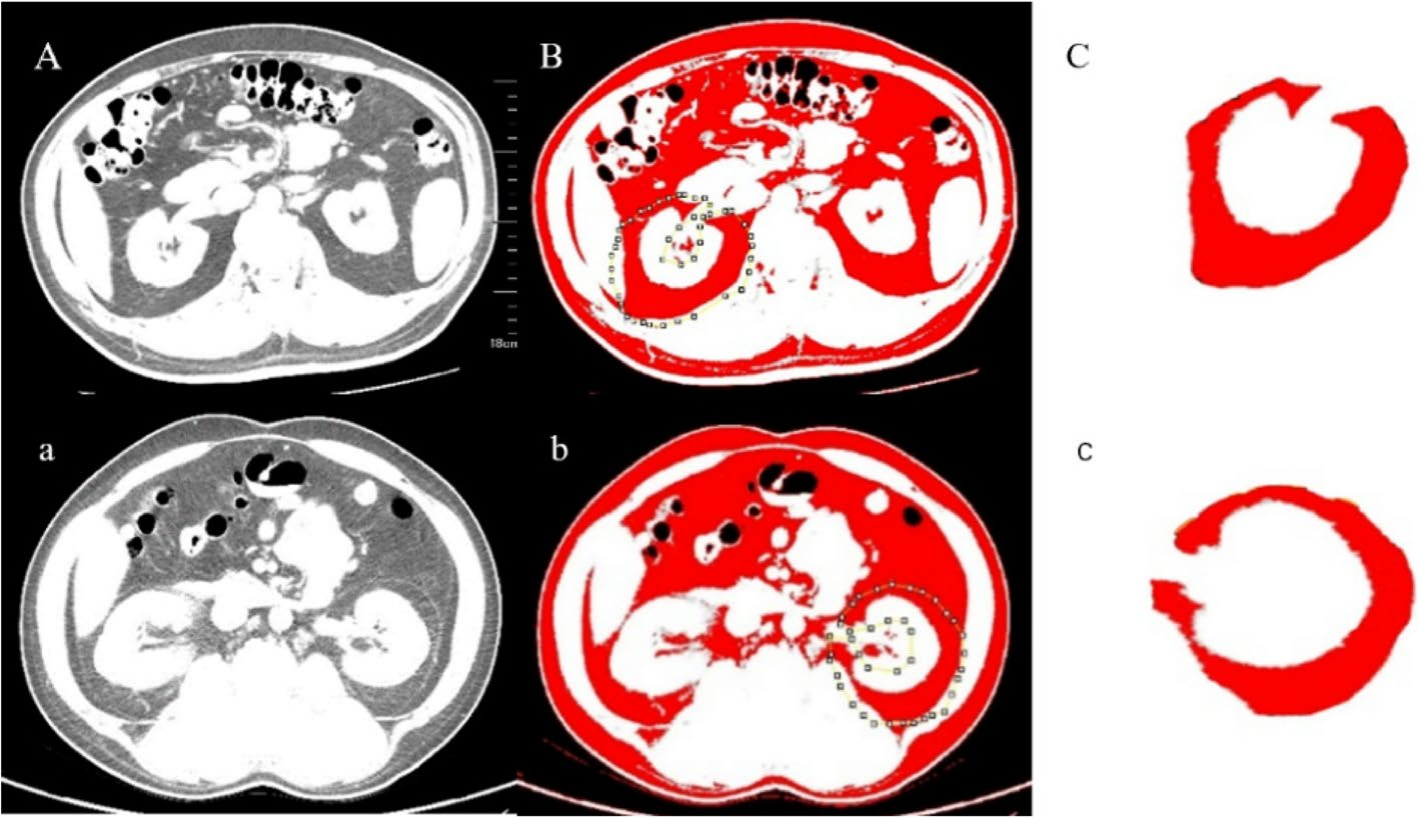
(A,a) CT images of the renal vein level; (B,b) outline the range of the perirenal fat on both sides; C: right perirenal fat area; c: left perirenal fat area

The PFA was defined as the region on axial CT images at the renal vein level, from the anterior renal fascia (Gerota’s fascia) to the lateral conical ligament surrounding the posterior perirenal fat (including the retroperitoneal fat posterior to the renal fascia, also known as Zuckerkandl’s fascia) [10] (Fig. 1). The PFA was measured using ImageJ 1.52a software by selecting and measuring this region on the CT images. Fat tissue was calculated using a standard HU range of − 190 to − 30 HU. The measurement of fat area was performed by two radiologists who were unaware of the patients'pathological results, And the mean value was used as the final outcome.

**Fig. 2:**
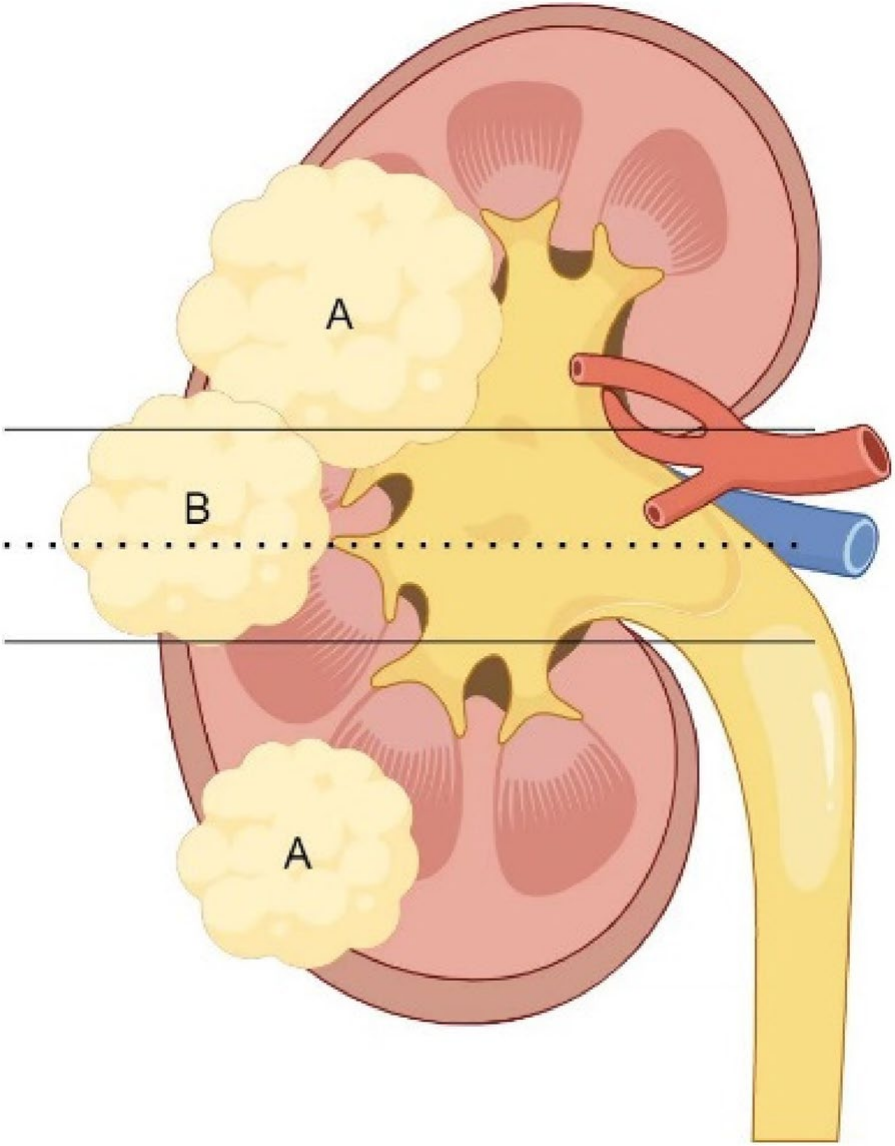
**A** The tumor is located outside the polar lines of the kidney (OPLK). **B** The tumor is located within the polar lines of the kidney (WPLK). Solid line: Pole line; Imaginal line: Center line

Definition of tumor location: The polar line consists of two parallel lines at the renal hilum. According to the relationship between tumor location and the polar line in the RENAL scoring system [11], we define tumors as being located within the polar lines of the kidney (WPLK) (3 points) if more than 50% of the tumor is within the polar lines, crosses the midline, or is entirely between the two polar lines (Fig. 2). Conversely, tumors are defined as being located outside the polar lines of the kidney (OPLK) (1–2 points) if less than 50% of the tumor is within the polar lines or if the entire tumor is outside the polar lines. Among them, six patients had large tumors that crossed the two polar lines and extended to one pole of the kidney, making it difficult to classify them clearly. Therefore, we excluded the data of these six patients in subsequent studies related to tumor location.

### Statistical analysis

All analyses were performed using Empower Stats software (version 4.1, X&Y Solutions, Boston, MA) and R software (version 4.2.2). Categorical variables are displayed as N (%). Continuous variables conforming to a normal distribution were presented as mean ± standard deviation (SD) and analyzed using the Student's t-test. Non-normally distributed data were expressed as median and range (Q1, Q3) and analyzed with the Mann–Whitney U test. Categorical variables were expressed as frequency counts and percentages (*N* [%]) and between-group differences were assessed using the chi-square (χ^2^) test. A t-test was also used to evaluate the relationship between PFA and tumor location. A Pearson's test was conducted to examine the correlation between PFA and BMI. Multivariate logistic regression analyses were conducted to examine the relationship between PFA and RCC pathological types, with results reported as odds ratios (OR) along with 95% confidence intervals (95% CI). To further assess the stability of the relationship between PFA and the pathological types of RCC, we constructed three stepwise adjusted regression models: Model 1 did not account for confounding factors; Model 2 adjusted for tumor side, BMI, height, and cPFA based on Model 1; and Model 3 further adjusted for hypertension, tumor size, diabetes, and pathological stage 1 based on Model 2.

## Results

**Fig. 3:**
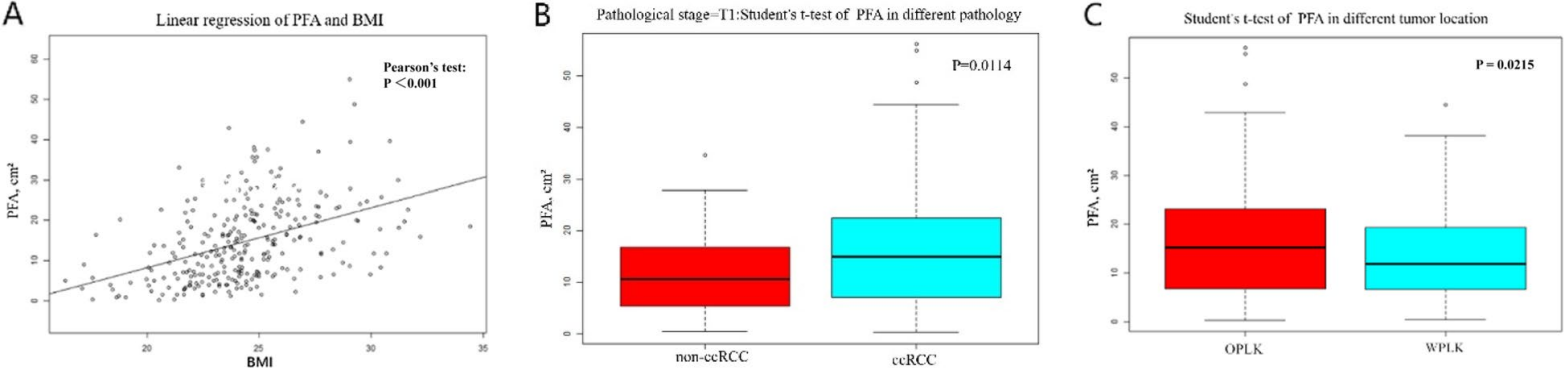
**A** Pearson's test revealed a linear relationship between PFA and BMI (*P* < 0.001). **B** The Student’s t-test demonstrated a significant association between PFA and tumor location (*P* = 0.0215) **C** The PFA of patients with tumors located in the OPLK is significantly higher than that of patients with tumors located in the WPLK. OPLK: Outside the polar lines of the kidney; WPLK: Within the polar lines of the kidney

This study included a total of 297 patients with RCC, of which 236 were ccRCC patients and 61 were non-ccRCC patients. Among 61 patients with non-ccRCC, the detailed histopathological classification is as follows: 27 cases of Papillary RCC, 10 cases of Chromophobe RCC, 5 cases of Clear cell papillary RCC, 9 cases of RCC Associated with Xp11.2 Translocation/TFE3 Gene Fusion, 2 cases of Sarcomatoid RCC, 1 case of Collecting duct RCC, and 7 cases of Unclassified RCC.The ccRCC group exhibited higher PFA (*p* = 0.007), cPFA (*p* = 0.01), weight (*p* = 0.002), BMI (*p* < 0.001), and incidence of T1 stage (*p* = 0.01). In contrast, the non-ccRCC group showed a higher incidence of T2 stage (*p* = 0.002) (Table 1). Pearson's test revealed a linear relationship between PFA and BMI (*P* < 0.001). The Student’s t-test demonstrated a significant association between PFA and tumor location (*P* = 0.0215), with patients whose tumors were located OPLK having a significantly higher PFA than those whose tumors were located WPLK. This is caused by selecting renal venous level CT images when measuring PFA. Considering that larger tumors may affect PFA, we conducted a stratified analysis based on pathological staging. The results showed that when the tumor is at stage T1, patients with ccRCC have a significantly larger PFA compared to those with non-ccRCC (Fig. 3).

**Table 1:** General baseline characteristics of participants

	N (%)	ccRCC	non-ccRCC	*P* Value
All participants	297	236	61	
Gender				0.483
Male	201 (67.68%)	162 (68.64%)	39 (63.93%)	
Female	96 (32.32%)	74 (31.36%)	22 (36.07%)	
Age(year)	58.03 ± 13.19	58.58 ± 12.76	55.89 ± 14.64	0.203
PFA (cm^2^)	14.90 ± 10.24	15.72 ± 10.44	11.76 ± 8.79	0.007^*^
cPFA (cm^2^)	15.04 ± 10.41	15.66 ± 10.51	12.59 ± 9.72	0.011^*^
Weight(kg)	66.91 ± 10.75	67.84 ± 10.57	63.34 ± 10.77	0.002^*^
Height(cm)	165.57 ± 8.08	165.78 ± 8.04	164.75 ± 8.25	0.399
BMI	24.33 ± 2.87	24.65 ± 2.84	23.14 ± 2.68	< 0.001^*^
Size(cm) M (Q1, Q3), cm	4.50 (2.80, 5.90)	4.38 (2.80, 5.50)	4.99 (3.00, 6.60)	0.069^#^
Tumor location				0.824
OPLK	176 (59.26%)	142 (60.17%)	34 (55.74%)	
WPLK	115 (38.72%)	89 (37.71%)	26 (42.62%)	
unclassifiable	6 (2.02%)	5 (2.12%)	1 (1.64%)	
Tumor side				0.944
Right	157 (52.86%)	125 (52.97%)	32 (52.46%)	
Left	140 (47.14%)	111 (47.03%)	29 (47.54%)	
Pathological stage 1				0.010^*^
T1a	157 (52.86%)	131 (55.51%)	26 (42.62%)	
T1b	94 (31.65%)	76 (32.20%)	18 (29.51%)	
≥ T2	46 (15.49%)	29 (12.29%)	17 (27.87%)	
Pathological stage 2				0.002^*^
T1	248 (83.50%)	205 (86.86%)	43 (70.49%)	
≥ T2	49 (16.50%)	31 (13.14%)	18 (29.51%)	
Hypertension				0.856
Yes	143 (48.15%)	113 (47.88%)	30 (49.18%)	
No	154 (51.85%)	123 (52.12%)	31 (50.82%)	
Diabetes mellitus				0.088
Yes	51 (17.17%)	45 (19.07%)	6 (9.84%)	
No	246 (82.83%)	191 (80.93%)	55 (90.16%)	

^*^P < 0.05

^#^Mann–Whitney rank sum test

We constructed three logistic regression models to investigate the independent effect of PFA (Table 2). Without adjusting for relevant covariates, PFA was significantly associated with RCC pathology [OR 1.05, 95% CI (1.01, 1.08), *P* = 0.0078], indicating that each 1 cm^2^ increase in PFA corresponds to a 5% increase in the probability of ccRCC pathology. However, in the models adjusted for covariates, the association between PFA and renal cell carcinoma pathology was not significant in either Model 2 or Model 3.

**Table 2:** Association between PFA with RCC pathological types

Exposure	Model 1 (OR,95%CI, P)	Model 2 (OR,95%CI, P)	Model 3 (OR,95%CI, P)
PFA	1.05 (1.01,1.08) 0.0078	1.05 (0.99,1.11) 0.1088	1.05 (0.98,1.11) 0.1596

Model 1: No covariate was adjusted

Model 2 adjusted for: tumor side, BMI, height, contralateral PFA

Model 3 adjusted for: tumor side, contralateral PFA, pathological stage 1, pathological stage 2. BMI, height, size, Diabetes mellitus

Given the significant correlation between PFA and tumor location and pathological staging in RCC patients, we performed a stratified analysis (Table 3). For patients with tumors located OPLK, a significant association between PFA and RCC pathology was observed. Further stratification by tumor location and pathological stage showed that in T1 stage patients with tumors located OPLK, PFA remained significantly associated with RCC pathology [OR 1.20, 95% CI (1.06, 1.37), *P* = 0.0045]. The probability of a ccRCC diagnosis increases by 20% for T1 stage patients with tumors located at OPLK, for each 1 cm^2^ increase in PFA.

**Table 3:** Subgroup analysis for the association between PFA and RCC pathological types

Model	Pathological stage 2 = T1	Pathological stage 2 ≥ T2	Total
Tumor location OPLK			
Model 1 (OR,95%CI, P)	**1.06 (1.01,1.11) 0.0300**	1.05 (0.96,1.14) 0.3178	**1.05 (1.01,1.10) 0.0169**
Model 2 (OR,95%CI, P)	**1.20 (1.06,1.36) 0.0043**	1.04 (0.87,1.22) 0.6568	**1.14 (1.04,1.26) 0.0057**
Model 3 (OR,95%CI, P)	**1.20 (1.06,1.37) 0.0045**	1.02 (0.85,1.22) 0.8297	**1.14 (1.04,1.26) 0.0070**
Tumor location WPLK			
Model 1 (OR,95%CI, P)	1.03 (0.97,1.10) 0.3359	1.00 (0.89,1.13) 0.9933	1.02 (0.97,1.08) 0.3793
Model 2 (OR,95%CI, P)	0.95 (0.87,1.05) 0.3147	0.00 (0.00, Inf) 0.9987	0.98 (0.90,1.07) 0.6677
Model 3 (OR,95%CI, P)	0.95 (0.86,1.05) 0.2998	Inf. (0.00, Inf) 0.9993	0.98 (0.90,1.07) 0.6807
Total			
Model 1 (OR,95%CI, P)	1.05 (1.01,1.09) 0.0197	1.03 (0.96,1.11) 0.3989	**1.04 (1.01,1.08) 0.0146**
Model 2 (OR,95%CI, P)	1.05 (0.98,1.13) 0.1808	1.08 (0.95,1.24) 0.2447	**1.05 (0.99,1.12) 0.1093**
Model 3 (OR,95%CI, P)	1.05 (0.98,1.13) 0.1862	1.07 (0.93,1.23) 0.3606	**1.05 (0.99,1.12) 0.1281**

Model 1: No covariate was adjusted

Model 2 adjusted for: tumor side, contralateral PFA, BMI, size

Model 3 adjusted for: tumor side, contralateral PFA, BMI, size, Diabetes mellitus

## Discussion

Obesity is a risk factor for RCC [12, 13], and ccRCC is the most common subtype of RCC. Studies have demonstrated a significant association between obesity and the occurrence and progression of ccRCC [14, 15]. Wang et al. [16] found that visceral fat area (VFA) could substitute BMI as a risk factor for ccRCC, noting that patients with elevated VFA levels have a higher occurrence of ccRCC compared to other RCC histological subtypes. Hu et al.'s [17] research demonstrated a connection between visceral fat and high-grade Fuhrman nuclear grading in ccRCC. Perirenal fat is considered a part of visceral fat tissue. Li S et al. [18] were the first to employ deep-learning algorithms to investigate the relationship between PFA and pathological grade in ccRCC patients, and discovered that perirenal fat provides incremental value in predicting the pathological grading of ccRCC. However, that study did not examine the association between PFA and pathological subtypes of RCC. Our findings demonstrated that ccRCC was associated with a higher PFA compared to non-ccRCC, and we additionally observed correlations between ccRCC and increased contralateral PFA, BMI, and body weight. For T1 stage patients with tumors located OPLK, high PFA is significantly correlated with ccRCC [OR 1.20, 95% CI (1.06, 1.37), *P* = 0.0045]. Perirenal adipose tissue may be associated with the occurrence and progression of ccRCC.

We found that some previous studies on perirenal fat tissue used perirenal fat thickness (PRFT) to assess fat content. In patients with type 2 diabetes, higher PRFT is connected to chronic kidney disease and heightens the risk of both cardiovascular and atherosclerotic cardiovascular diseases [18, 19]. There are few studies on the correlation between PRFT and RCC. Higher PRFT predicts poorer progression-free survival (PFS) in patients with localized ccRCC [20]. However, recent studies have shown that PRFT increases overall survival (OS) and PFS in metastatic RCC patients treated with antagonists of vascular endothelial growth factor (anti-VEGF). This may be related to the fact that tumors in patients with higher PRFT exhibit increased angiogenic features [21]. Perirenal fat thickness is a one-dimensional linear measurement, whereas PFA represents a two-dimensional parameter. We propose that PFA is a more suitable metric for assessing perirenal fat deposition.

Obesity can lead to abnormal expression of adipokines, a chronic inflammatory state, and insulin resistance, all of which contribute to the occurrence and progression of renal RCC [22, 23]. However, BMI does not reflect the abnormal distribution of fat. Increasing evidence suggests that the relationship between visceral fat and RCC is more closely associated. Visceral fat tissue is considered an endocrine organ, with a rich vasculature, nerve innervation, and an abundance of inflammatory cells. Visceral fat is capable of releasing different hormones and cytokines that are linked to cancer development and tumor growth, and it can also enhance the expression of genes associated with tumor cell invasion and spread [24–26].

The origin of perirenal fat is preadipocytes and mature adipocytes, setting it apart from other visceral fat tissues and explaining its specific function [24, 27]. Perirenal fat tissue covers the surface of the kidneys and is more metabolically active in terms of fat metabolism and adipokine secretion compared to other visceral fat. This may directly contribute to the occurrence and progression of kidney diseases [18, 28]. A study on animals demonstrated that perirenal fat directly led to endothelial dysfunction in the renal artery, partially through the action of tumor necrosis factor-α [29]. Sanchez et al. [27] also found that the fat tissue surrounding RCC tumors created hypoxic regions and an inflammatory state, promoting angiogenesis and changes in the tumor microenvironment, thereby advancing RCC progression. Research indicates that ccRCC causes the browning of nearby perirenal fat, which aids in tumor growth, invasion, and metastasis through lactate secretion [30]. In conclusion, there is a complex interaction between perirenal fat and RCC, especially ccRCC. This also supports our research conclusion: a higher perirenal fat area is associated with ccRCC. In clinical practice, PFA assists in preoperative prediction of RCC pathological subtypes, thus guiding clinical treatment strategy decisions.

The study has some limitations. First, it is a retrospective study. Second, we included cases from two centers, but one center had a relatively small sample size. Since the two centers are located in the same region and the population characteristics are nearly identical, we combined the data for statistical analysis. Third, due to the use of renal venous CT scan images to measure PFA, the measurement of PFA may be significantly affected in patients with tumors located within the polar lines or with larger tumors, as the tumor compresses the surrounding fat. Fourth, the current analysis is constrained by the limited cohort size of the non-ccRCC group (n = 61), which may limit the generalizability of our results. Therefore, in our study, we performed stratified analysis based on tumor location and pathological staging to mitigate the impact of these factors.

## Conclusions

Our research findings show that a higher PFA is associated with renal ccRCC. PFA may serve as a predictive marker for ccRCC. This study not only confirms that perirenal fat is a risk factor for ccRCC, but also suggests that perirenal fat may promote the occurrence and progression of ccRCC, the underlying mechanisms of which require further investigation.

## Data Availability

The datasets used and/or analysed during the current study are available from the corresponding author on reasonable request.

## Abbreviations

RCC: Renal cell carcinoma
ccRCC: Clear cell renal cell carcinoma
non-ccRCC: Non-clear cell renal cell carcinoma
CT: Computed tomography
PFA: Perirenal fat area
OPLK: Polar lines of the kidney
BMI: Body mass index
cPFA: Contralateral perirenal fat area
WPLK: Within the polar lines of the kidney
OR: Odds ratios
95% CI: 95% Confidence intervals
VFA: Visceral fat area
PRFT: Perirenal fat thickness
PFS: Progression-free survival
OS: Overall survival
anti-VEGF: Antagonists of vascular endothelial growth factor
